# Everybody's Page

**Published:** 1892-08-27

**Authors:** 


### EVERYBODY'S PAGE.

An attempt was, we believe, made some years ago to show that as there is an enormous loss of chlorides from people who are attacked by cholera, this disease could be effectively controlled by administering chlorides in abundance. Experience has proved the fallaciousness of this supposition. But in hot weather salt can advantageously be taken in much larger quantities than usual, as during the heat of the summer the system naturally loses a large quantity of salt.

A new remedy for warts has been found, which is not likely to come into extensive use, or to take its place in any pharmacopœia. Nevertheless we give it, as it is unfair to deprive Americans of the honour and glory to be gleaned from a discovery, even though it will not revolutionize an effete civilization. Briefly, the remedy is the blood of the porpoise. It is related that a man whose hands were covered with warts, which declined to succumb to the blandishments of every conceivable treatment, shot a porpoise in the West Indies, and, in cutting up the carcass his hands were besmeared with blood. The result was magical; the warts disappeared with an alacrity which made full amends for the stubbornness which they had previously shown, and were obliging enough not to return.

The result of the investigations made by Dr. Tassinari on the influence of tobacco smoke on the germs of cholera, will be gratifying to those smokers who uphold the use as well as pleasure of the weed which is so noxious in the eyes of some. The doctor's investigations were not confined to cholera, but were extended also to anthrax, pneumonia, and typhoid. With the cholera germ the influence of tobacco smoke proved remarkable. The method of procedure was to line the interior of hollow balls with gelatine containing germs of the various diseases experimented on; tobacco smoke was then passed through the balls for a period varying from ten to thirty minutes. At the expiration of this time the bacilli of Asiatic cholera and of pneumonia were found to be completely destroyed. The effect on the anthrax bacillus was not so destructive, and the bacillus of typhoid refused to succumb to the charms of tobacco.

The anti-spasmodic power of sulfonal appears to be of greater use than its sleep-producing power. A Chicago physician reports a case of painful cramps in a fractured femur which were successfully treated with this drug, the action of which proved slow but efficacious.

Those who look with such aversion upon the drink bill of the English nation will find little comfort in the perusal of the Report of the Commissioners Customs. Despite their well-directed efforts the bill increases; yet some satisfaction is to be gleaned from the fact that crime, which is popularly supposed to have such close connection with alcohol, shows signs of decreasing.

An experiment is being made in New York during the hot season in the way of providing sterilised milk for the poor in the neighbourhood of the Good Samaritan Dispensary. The idea originated with some ladies who, for some time past have been endeavouring to teach the tenement house population of the city that something can be done for the protection of infant life against some of the germ diseases by which it is attacked in hot weather. The project appears to be one of a purely charitable nature, without any chance of proving a success commercially, as the sterilised milk is sold under cost price, in order that it may compete with ordinary milk.

A novel use has been discovered for cork legs. Hitherto they have been viewed in the unromantic light of aids to the propulsion of unfortunate people who have lost part of their means of locomotion. A career of extended utility has been found for them as a means of tracing people's whereabouts by a gentleman evidently of an original, if not Hibernian, turn of mind, who wrote recently to a provincial postmaster: "May I ask you to be good enough to let one of your carriers take the enclosed post card to my nephew. He is a young man, I believe, well known in your town, but whose address I forget (if ever I knew it). He walks lame owing to a cork leg, has also a bright, projecting set of teeth." The town in which the young man lived containde 72,000 inhabitants!

